# Effect of Arm Eccentric Exercise on Muscle Damage of the Knee Flexors After High-Intensity Eccentric Exercise

**DOI:** 10.3389/fphys.2021.661618

**Published:** 2021-04-08

**Authors:** Xin Ye, William M. Miller, Sunggun Jeon, Jun Seob Song, Tyler J. West

**Affiliations:** ^1^Department of Rehabilitation Sciences, University of Hartford, West Hartford, CT, United States; ^2^Department of Health, Exercise, and Recreation Management, University of Mississippi, University City, MO, United States; ^3^School of Kinesiology, Applied Health and Recreation, Oklahoma State University, Stillwater, OK, United States

**Keywords:** eccentric muscle contraction, muscle damage, crossover, delayed onset muscle soreness, range of motion, maximal isometric contraction strength, recovery

## Abstract

Repeated bout effect (RBE) describes a phenomenon that an initial unaccustomed eccentric exercise (ECC) bout can confer a protective effect against muscle damage from the subsequent same exercise. This protection has been observed in the same muscle, as well as the contralateral homologous (CL-RBE) muscle. But it is unknown whether the RBE is evident for non-local unrelated heterogonous muscles. The purpose of this study was to examine whether an initial elbow flexion (EF) muscle-damaging ECC could confer RBE against muscle damage from the subsequent ECC performed in the remote lower limb knee flexor (KF) muscle group. Twenty-seven young individuals were randomly assigned into the experimental (EXP: *n* = 15) and the control (CON: *n* = 12) groups. All participants performed a baseline unilateral KF ECC (six sets of 10 repetitions) on a randomly chosen leg. After a washout period (4 weeks), the EXP group performed 60 high-intensity unilateral EF ECC on a randomly chosen arm, followed by the same intensity exercise using the contralateral KF muscle group 2 weeks later. The CON group performed the same contralateral KF ECC, but with no prior EF ECC bout. Changes in the KF muscle damage indirect markers (muscle soreness, range of motion, and maximal isometric strength) after the ECC were compared between the baseline and second bouts for both groups with mixed factorial three-way (group × bout × time) ANOVA. Additionally, index of protection for each damage marker was calculated at 1 and 2 days after the ECC and compared between groups with independent *t*-tests. For both groups, the magnitude of the changes in the damage markers between the baseline and the second ECC bouts were not significantly different (all values of *p* > 0.05). As for the index of protection, relative to the CON, the EXP showed an exacerbating damaging effect on the KF isometric strength following the second ECC bout, particularly at the 1-day post-exercise time point (index of protection: EXP vs. CON mean ± SD = −29.36 ± 29.21 vs. 55.28 ± 23.83%, *p* = 0.040). Therefore, our results do not support the existence of non-local RBE.

## Introduction

High-intensity resistance exercise training is widely used in a variety of populations for athletic and rehabilitative purposes. With both concentric (shortening) and eccentric (lengthening) muscle contractions involved, high-intensity resistance exercise often induces temporary performance decrements on the neuromuscular system, featuring muscle fatigue and muscle soreness (Ye et al., 2014, 2015; Beck et al., 2016). It is also believed that the lengthening (eccentric) portion of the dynamic muscle contractions is primarily responsible for the micro-damage in the skeletal muscle fibers, especially when individuals are novice to such exercise. The eccentric exercise-induced muscle damage results in prolonged symptoms such as decreased strength, delayed-onset muscle soreness (DOMS), decreased range of motion (ROM), and increased creatine kinase and myoglobin concentrations in the blood (Warren et al., 1999). Interestingly, it has been well-documented that skeletal muscles possess a protective mechanism (Hyldahl et al., 2017; Chen et al., 2019): while an initial unaccustomed high-intensity eccentric exercise (ECC) bout can induce tremendous amount of muscle damage, the magnitude of muscle damage is usually attenuated in the subsequent bouts of the same exercises. This phenomenon is referred to as the repeated bout effect (RBE; Nosaka and Clarkson, 1995). To this day, several possible mechanisms of the RBE have been identified, including neural adaptations, altered inflammatory sensitivity, muscle-tendon complex adaptations, and muscle extracellular matrix remodeling (see details in Hyldahl et al., 2017 for review). However, the exact mechanisms of the RBE are unknown, and whether these mechanisms work independently or together to provide the protection is still not clear.

In addition to the widely observed RBE from the same muscle group undergoing repeated muscle-damaging exercise bouts, research studies (Connolly et al., 2002; Howatson and van Someren, 2007; Starbuck and Eston, 2012; Chen et al., 2016, 2018) have also observed the protective effect on the remote contralateral limb after an initial unilateral damaging exercise, known as the contralateral RBE (CL-RBE). While several mechanisms contribute to the RBE, the possible mechanisms of the CL-RBE are only likely mediated by neural and inflammatory adaptations (Hyldahl et al., 2017), because the contralateral muscle is not exposed to the initial bout of ECC, thus not receiving direct mechanical stimulus. Regarding neural mechanism, the adaptations have been observed in different sites of the nervous system. For example, increased muscle fiber activation of the contralateral muscle was evidently shown for the contralateral exercise bout (Tsuchiya et al., 2018). Additionally, greater cortical (contralateral non-exercised side) level modulations have also been observed after ECC in both acute and chronic cross education studies (Howatson et al., 2011; Kidgell et al., 2015), when compared to the concentric exercise. As for the adaptations of the inflammatory response, Xin et al. (2014) conducted a muscle biopsy study to examine contralateral RBE in the knee extensor muscles. Specifically, biopsy samples were analyzed from both the ipsilateral and contralateral knee extensor muscles before and after two bouts of ECC (separated by 4 weeks), and the authors found that the attenuation of increased nuclear-factor kappa B (a muscle inflammation regulator) activity in the contralateral muscle at the second bout, as compared to that at the first bout. This indicates the attenuated inflammatory signaling on the second bout when the contralateral muscle is exposed to ECC. However, it is not entirely clear whether the modulation of contralateral muscle nuclear-factor kappa B was mediated through the neural pathway, or the circulatory pathway. If the latter is the case, then it is also expected to see RBE in non-local unrelated heterogonous muscles due to circulation (e.g., an initial arm ECC confers protective effect against muscle damage from the subsequent leg ECC, or vice versa).

In the recent decade, there has been an emerging interest in investigating the non-local global effects of unilateral exercise on the remote unrelated non-exercised heterogonous muscle function and performance, along with the traditionally studied contralateral crossover effects research. The specific term “non-local” is used to describe this line of research, such as non-local exercise on muscle fatigue (Halperin et al., 2015; Doix et al., 2018; Ye et al., 2018; Miller et al., 2019) and non-local stretching on range of motion (ROM; Behm et al., 2021). Muscle damaging activities such as downhill running may also induce non-local effects in a remote unrelated heterogonous muscle group. For example, after having the participants perform a 1-h downhill running exercise, Brandenberger et al. (2019) found prolonged decrements in the elbow flexor (EF) muscle strength and voluntary activation (up to 2 days), but not the resting twitch force. Collectively, these interesting findings suggested that the downhill running-induced muscle damage can induce a prolonged central effect, influencing the performance of the remote upper limb muscles. However, limited information is available regarding the potential non-local RBE from performing an initial muscle damaging ECC in a remote unrelated heterogonous muscle group.

Therefore, we conducted this pilot study mainly to examine whether an initial unilateral ECC would confer a protective effect on muscle damage from performing a subsequent similar exercise in a remote unrelated heterogonous muscle group. There was a very recent study showing that no protective effect on maximal EF eccentric contractions was conferred by prior lower limb (knee flexion or knee extension) eccentric exercises (Chen et al., 2021). Thus, we chose to have the initial ECC bout performed in the EF muscles, and to have the second ECC performed in the knee flexor (KF) muscles. The results of this study could provide important information, because it adds the information to the literature of RBE, and it may also help better understand the underlying mechanisms of the RBE.

## Materials and Methods

### Participants

Since no study has examine the potential non-local RBE from the EF on the KF, we estimated the sample size based on the data from both Chen et al. (2016, 2018), where the authors examined the CL-RBE of EF and KF. Based on the effect size of 1 for a possible difference in maximal isometric strength changes between the ipsilateral and contralateral conditions, it was shown that at least 11 participants per group were necessary for the comparisons between baseline and the second bout, with the alpha level of 0.05 and power (1−β) of 0.80 (Cohen, 1988) by G*Power (G*Power 3.1.9.4, Heinrich-Heine-Universitat Dusseldorf, Dusseldorf, Germany; Beck, 2013). Twenty-nine individuals were recruited into this study, with 15 assigned into the Experimental group (EXP) and 14 assigned into the Control group (CON). Two participants from the CON dropped out from the experiment, thus a total of 27 individuals (EXP: six men and nine women, mean ± SD: age = 21 ± 3 years, height = 170.0 ± 8.5 cm, body weight = 70.3 ± 14.2 kg; CON: seven men and six women, age = 22 ± 3 years, height = 172.8 ± 10.3 cm, body weight = 73.3 ± 29.1 kg) completed this study. All participants were healthy and had not performed any regular resistance or aerobic training regimen in the past 1 year prior to this study. In addition, their daily life did not include activities such as carrying heavy objects frequently. Prior to any experimental testing, each participant completed an informed consent and a pre-exercise health and exercise status questionnaire, which indicated no current or recent (1 year) neuromuscular or musculoskeletal disorders in both the upper and lower extremities. During the consenting process, the participants were instructed to maintain their normal habits in terms of dietary intake, hydration status, and sleep, and to refrain from vigorous physical activities during the entire study. This study was approved by the University Institutional Review Board (Approval Code: 19-025), and was conducted in conformity with the policy statement regarding the use of human subjects by the Declaration of Helsinki.

### Study Design

**Figure 1:**
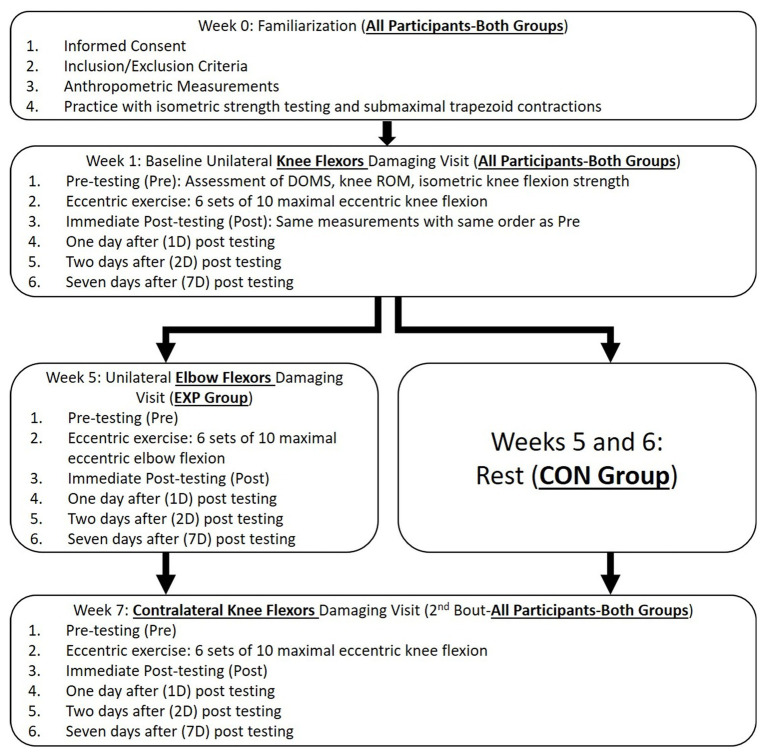
Study design and the procedure flowchart of the experiment. DOMS: delayed onset muscle soreness; ROM: range of motion; EXP: experimental group; and CON: control group.

The main purpose of this study was to examine whether performing an initial ECC in an upper limb muscle group (elbow flexors) could induce any protective effect against the muscle damage from the subsequent ECC performed in a lower limb muscle group (KFs). Thus, a between-group repeated-measures design was used. Figure 1 shows the flowchart of the experimental design of this study. A baseline ECC damaging protocol on the KF muscles was conducted on both EXP and CON groups (Week 1). Specifically, the baseline ECC was performed with a randomly chosen leg (either dominant or nondominant leg). The choice of the dominant and nondominant legs was counterbalanced among the participants (dominant: *n* = 14; nondominant: *n* = 13). This setup (baseline damaging bout) specifically allowed us to record the responses of the indirect markers of the KF muscle damage, and it served as a reference to calculate the index of protective effect. Ideally, the exact same muscle would be used in the baseline and the second ECC bouts. However, due to that the RBE can last longer than 6 months (Nosaka et al., 2001), the washout period would not fit the experiment timeline, we therefore chose to have the participants perform the second ECC bout in the contralateral KF. According to Chen et al. (2018), the CL-RBE was evident for KF muscles, but disappeared between 7 and 28 days. Using this time course information, we specifically arranged a 6-week washout period between the baseline unilateral KF ECC and the second bout contralateral KF ECC. Such design also allows the comparison between the magnitudes of the CL-RBE (from the CON group, if any) and non-local RBE (from the EXP Group). At least 72 h after the familiarization visit, with a 4-week rest period after the baseline ECC, only the EXP returned to the laboratory at the beginning of Week 5 for the ECC on a randomly chosen EF muscle group (either dominant or nondominant arm). Lastly, the second bout of ECC was performed by both EXP and CON groups at the beginning of Week 7, during which the contralateral KF was damaged by the identical exercise as performed during Week 1. Before (Pre), immediately (Post), 1 day (1D), 2 days (2D), and 7 days (7D) after all the ECC sessions, indirect muscle damage markers were measured. For each participant, effort was made to ensure that the eccentric exercises and experimental tests were conducted roughly the same time during each visit. The leg and arm dominance were determined based on which foot the participants would kick a ball and which hand they would throw a ball, respectively.

### Familiarization

A familiarization session (Week 0) was scheduled before the baseline testing week. The purposes of this session were (1) to familiarize the participants with the experimental measurements (muscle soreness, range of motion, and isometric strength testing); (2) to test the participants’ 1-repetition maximum (1RM) strength values for both KF and EF muscle groups; and (3) to have the participants practice ECC. The participants were introduced to the visual analog scale (VAS), the ROM testing procedures, and then were instructed to practice maximal voluntary isometric contractions (MVICs) on the custom-built isometric strength testing stations for both KF and EF muscle groups. After the familiarization with these testing measurements, the 1RM strength testing for KF was conducted with a leg curl machine (ProClub Line Leverage Leg Curl; Body-Solid Inc., Forest Park, IL, United States), followed by EF 1RM strength test with a dumbbell on a preacher curl bench (CB-6 Adjustable Arm Curl Bench; Valor Fitness United States, Seminole, FL, United States). With the proper and comfortable body position on the exercise machines, the test started with the participants performing a warm-up set of 5–10 repetitions using approximately 50% of the estimated 1RM. After an adequate 1–2 min of rest period, a set of 2–5 repetitions was performed at about 75% of the estimated 1RM. Following another rest, the participants performed the first actual 1RM attempt. The 1RM was finally determined within 3–5 attempts after the warm-up. The minimal detectable increment/decrement of the 1RM values were 1.14 and 0.45 kg for the KF and EF, respectively. Lastly, the participants practiced the ECC on the exercise machines for the designated muscle group(s) (EXP: both KF and EF muscle groups; CON: KF muscle group only) by following a 2-s up/2-s down tempo produced by a smartphone app (Pro Metronome; EUMLab, Berlin, Germany). No external load was added during the ECC practice.

### Eccentric Exercise

All participants performed the baseline (at the beginning of Week 1) ECC bout in the designated KF muscle group. The exercise protocol consisted of six sets of 10 ECC leg curls with the load equivalent to 150% of the leg curl 1RM. Prior to each set, the participants lay prone on the platform of the leg curl machine, with both ankles placed underneath the lever foam pad (fully extended knee joint, 0° knee joint angle). A Velcro® strap was used to tie the exercised ankle with the lever pad firmly, so the participants’ exercised leg could be passively pulled up to the starting position (120° knee joint angle; full knee extension = 0°) of each ECC contraction. Following the tempo, the research staff moved up the leg curl lever pad to the starting position with 2 s, released the weight, and then the participants gradually lowered the ankle with 2 s and with the controlled manner. Immediately after each exercise set, the rating of perceived exertion (RPE) was recorded using the 20-point Borg Rating of Perceived Exertion Scale (Borg, 1998). A 2-min rest interval was provided between consecutive ECC sets, during which the participants could get off the leg curl machine to walk around. During the exercise, the research staff verbally encouraged the participants, and closely monitored the movement of the ECC contractions. If the participants could not lower the lever with the controlled manner, the exercise load would be decreased by 10% of the 1RM load for the subsequent set until the contraction manner returned to normal.

The EF ECC was performed by the EXP at the beginning of Week 5, during which the identical exercise protocol parameters (e.g., relative intensity, contraction tempo, and rest duration) were used as during the baseline ECC. Starting from approximately 120° elbow joint angle, the research staff handed a preloaded dumbbell to the participants, then the participants lowered the dumbbell to the end of the ROM (fully extended arm, 0° elbow joint angle) with the controlled manner.

The second bout KF ECC was performed at the beginning of Week 7, in the contralateral KF muscle groups. It is important to mention that the 1RM testing of the KF was performed on Week 5, following the standard 1-RM testing procedures, as mentioned previously. The second bout ECC was identical to the baseline exercise, with the matched exercise intensity throughout all six sets. For example, if the eccentric load was decreased to 140% of the 1RM strength for the last two sets during the baseline exercise (Week 1), then the load would be specifically adjusted to 140% of the 1RM for the last two sets of the second bout (Week 7) ECC.

### Indirect Markers of Muscle Damage

The indirect markers of muscle damage of this study included KF and EF muscle soreness, KF and EF ROM, and KF and EF isometric strength. The test-retest reliability for the indirect markers were calculated between the values from the familiarization session and the pre-exercise values from the baseline KF visit (for KF measurements), as well as between the values from the familiarization session and the pre-exercise values from the EF visit (for EF measurements) by determining the intraclass correlation coefficient (model “3,1”: ICC3,1; Weir, 2005) and coefficient of variation (CV; Hopkins, 2000). For both KF and EF, the ICC3,1 and CV were at least 1.00 and 0%, 0.93 and 4.2%, and 0.88 and 10.6% for muscle soreness, ROM, and isometric strength, respectively.

#### Muscle Soreness

Muscle soreness for both muscle groups was assessed using a 100-mm VAS. The VAS scale read “No soreness at all” on the left side and “Unbearable pain” on the right side. Immediately before this measurement, the participants were asked to flex and extend the tested muscle group forcefully throughout the entire ROM three times, and they were asked to mark a vertical line on the VAS scale at the location representing their current soreness level from the designated muscles.

#### Range of Motion

A 12-in and an 8-in manual goniometer (EMI Plastic Goniometer; Elite Medical Instruments, Fullerton, CA, United States) were used to measure knee joint and elbow joint angles, respectively. For the measurement of the KF ROM, the participants stood on a 2-in metal plate with the non-tested foot, so the tested limb was hanging down relaxed without touching the floor. The KF ROM was then measured as the difference between the knee joint angles of the maximal voluntarily flexion and the naturally relaxed extension. For the EF ROM, the participants were asked to maintain an upright standing position with both arms hanging naturally and relaxed. The elbow joint ROM was then measured as the difference between the elbow joint angles of maximal voluntarily flexion and the naturally relaxed extension. At least three trials with 15-s rest between trials were performed to measure the ROM. If the values from any two trials differed more than two degrees, then extra trials would be conducted. The average of the three closest trials was then calculated and recorded as the ROM (Killen et al., 2019).

#### KF and EF Isometric Strength

The KF and EF isometric strength tests were conducted on two different custom-built isometric strength apparatuses described in Jeon et al. (2019). Briefly, the participants were asked to contract the tested muscle against an immovable force transducer (Model SSM-AJ-500; Interface, Scottsdale, AZ, United States). The testing joint angles for the KF and EF were 0 (extended knee joint) and 90°, respectively. Before the actual testing, the participants were instructed to perform three isometric contractions at about 50% of the perceived maximal effort to warm up. Specifically, they were told to “squeeze as fast as possible,” as they practiced during the familiarization session. The participants then performed three, 3-s MVICs with a 1-min rest interval contractions. During each MVIC, the research staffs provided a verbal countdown “three, two, one, pull” to the participants, with specific emphasis on “pull as fast as possible and as hard as possible.” During all maximal contractions, the research staffs provided strong verbal encouragement. For each MVIC, the force signal was sampled at 2 kHz and stored in a laboratory computer (Dell XPS 8900, Round Rock, TX) for further analyses. The peak force output was determined from the highest 500-ms portion of the force plateau during the contraction of the 3-s MVIC. The isometric strength was then determined by averaging the peak force values from the three MVICs. In addition, the relative isometric strength values at the Post, 1D, 2D, and 7D after all ECC bouts were calculated as the percentages of the Pre-values (100%).

### Index of Protection

The index of protection for each group was calculated using the values at 1D and 2D after exercise for muscle soreness, ROM, and KF isometric strength. Specifically, the index was calculated by the following formula: [(The magnitude of change in a variable after the first bout exercise – the magnitude of change in a variable after the second bout exercise)/(The magnitude of change in a variable after the first bout exercise) × 100; Hyldahl et al., 2017].

### Statistical Analyses

Assumptions for normality of distribution were checked and confirmed using the Shapiro-Wilk test. Separate two-way [bout (baseline, second) × limb order (started with dominant limb, started with nondominant limb)] mixed factorial ANOVA were used to examine the pre-exercise values of dependent variables as well as the 1RM strength values between two exercised KFs at the baseline and second bouts of exercise. The RPE responses over the ECC sets between the baseline and the second bouts were also examined by a three-way [bout (baseline, second) × limb order (started with dominant limb, started with nondominant limb) × set (1–6)] mixed factorial ANOVA. For the EF muscle damage indirect markers, one-way [time (Pre, Post, 1D, 2D, and 7D)] repeated measures ANOVAs were used to examine the potential changes following the EF ECC. Additionally, separate three-way [bout (baseline, second) × group (EXP, CON) × time (ΔPost-Pre, ΔPost1D-Pre, ΔPost2D-Pre, and ΔPost7D-Pre)] mixed factorial ANOVAs were used to examine the magnitude of the changes in the dependent variables (muscle soreness, ROM, and KF relative isometric strength) between the baseline and second bouts. Lastly, the index of protection for each KF muscle damage indirect markers was also compared between the EXP and the CON groups by using independent *t*-tests. The partial *η*^2^ statistic is provided for all repeated measure comparisons, with values of 0.01, 0.06, and 0.14 corresponding to small, medium, and large effect sizes, respectively (Cohen, 1988). In addition, Cohen’s d is also calculated for all *t*-statistics, with 0.20, 0.50, and 0.80 as small, medium, and large effect sizes, respectively (Cohen, 1988). All statistical tests were conducted using statistical software (IBM SPSS Statistics 22.0, IBM, Armonk, NY) with alpha set at 0.05. All data are reported as mean ± SD, unless otherwise stated.

## Results

### Pre-exercise Measurements and RPE During ECC

Table 1 shows the pre-exercise (immediately before the ECC) values of the muscle damage indirect markers and the 1RM KF strength before both baseline and second KF ECC bouts. No significant differences in any of these pre-exercise values were observed between the both ECC bouts, regardless which limb started the baseline ECC bout. For the RPE during the KF ECC, the three-way ANOVA only showed a main effect for set (*F* = 69.967, *p* < 0.001, and partial *η*^2^ = 0.854). The pairwise comparisons indicated significant RPE value differences between any two sets (*p* < 0.05), showing that the RPE values were significantly increasing throughout all six ECC sets (mean ± SD: set 1: 13.6 ± 1.7; set 2: 14.4 ± 1.6; set 3: 15.6 ± 1.7; set 4: 16.3 ± 1.3; set 5: 17.0 ± 1.1; and set 6: 17.6 ± 1.0).

**Table 1:** Pre-testing values of the muscle damage indirect markers before both baseline and second knee flexion ECC bouts.

	Started with the dominant limb		Started with the nondominant limb	
	Baseline (Week 1)	Second bout (Week 7)	Baseline (Week 1)	Second bout (Week 7)
Muscle soreness-VAS (mm)	1.46 ± 2.60	1.43 ± 0.31	2.38 ± 4.09	1.73 ± 2.00
Range of motion (°)	103.11 ± 11.46	102.68 ± 9.88	97.65 ± 13.68	98.72 ± 7.60
Isometric strength (N)	224.53 ± 55.82	214.05 ± 67.12	217.86 ± 72.38	203.27 ± 58.02
1-RM concentric strength (kg)	65.18 ± 32.59	61.96 ± 31.09	61.73 ± 28.95	63.85 ± 28.53

### Changes in the EF Muscle Damage Indirect Markers

**Figure 2:**
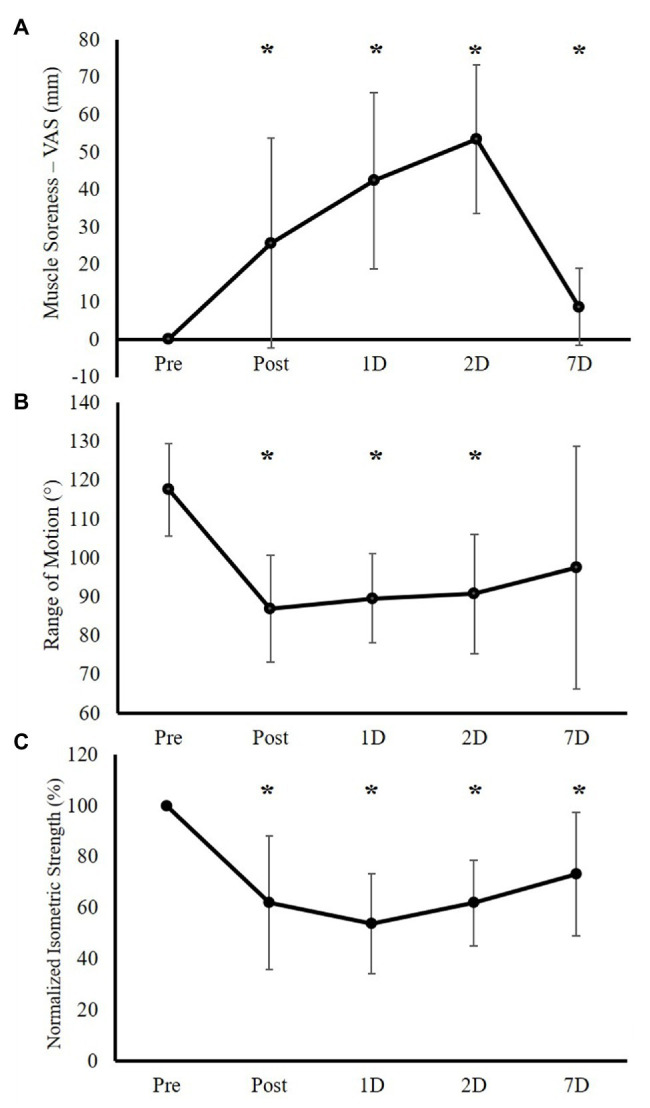
Elbow flexor muscle damage indirect markers’ responses immediately following (Post), 1 day after (1D), 2 days after (2D), and 7 days after (7D) the elbow flexion (EF) eccentric exercise (ECC) intervention (Experimental Group only, *n* = 15). **(A)** Muscle soreness responses as measured by visual analog scale (VAS). **(B)** Range of motion responses. **(C)** Maximal isometric strength responses (normalized as the percentage of the Pre-testing isometric strength). *Significant difference between Pre-testing value.

Figure 2 shows each EF muscle damage indirect markers responses following the EF ECC bout. Significant main effects for time were observed for all muscle damage indirect markers (all values of *p* ≤ 0.007).

### Comparisons of KF Muscle Damage Indirect Markers Responses Following the Baseline and Second KF ECC Bouts

**Figure 3:**
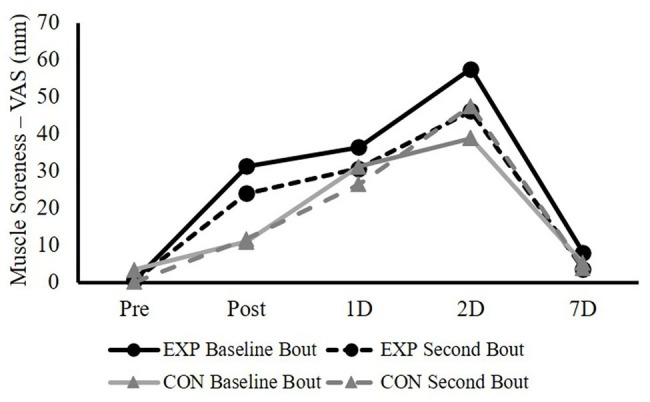
The responses of knee flexor (KF) muscle soreness (measured by VAS) immediately after (Post), 1 day after (1D), 2 days after (2D), and 7 days after (7D) the baseline and the second bouts of ECC for both experimental (EXP) and control (CON) groups.

Figure 3 shows both groups’ responses of muscle soreness (VAS) following both baseline and second bouts of ECC. For the magnitude of changes in muscle soreness, the three-way ANOVA showed significant bout × group × time (*F* = 2.740, *p* = 0.049, and *η*^2^ = 0.099) and bout × group (*F* = 6.291, *p* = 0.019, and partial *η*^2^ = 0.201) interactions. In addition, there was a main effect for time (*F* = 53.132, *p* < 0.001, and partial *η*^2^ = 0.680). Because the main point was to compare the indirect muscle damage markers’ responses following the baseline and the second bouts to determine the RBE, the follow-up tests were conducted using paired samples *t*-tests to compare the magnitude of changes in muscle soreness at each time point between the baseline bout and the second bout for both groups (EXP and CON), which did not indicate any significant differences for both groups (all values of *p* > 0.05; Table 2).

**Table 2:** The paired comparisons (*p*-value and Cohen’s d) of the magnitude of changes from the Pre-testing value in muscle soreness and maximal isometric strength at each time point between the baseline bout and the second bout for both EXP and CON groups.

	EXP: Baseline bout vs. Second bout				CON: Baseline bout vs. Second bout			
	Δ(Pos-Pre)	Δ(1D-Pre)	Δ(2D-Pre)	Δ(7D-Pre)	Δ(Pos-Pre)	Δ(1D-Pre)	Δ(2D-Pre)	Δ(7D-Pre)
Muscle soreness	*p* = 0.229 *d* = 0.30	*p* = 0.230 *d* = 0.28	*p* = 0.062 *d* = 0.52	*p* = 0.132 *d* = 0.54	*p* = 0.246 *d* = 0.33	*p* = 0.544 *d* = 0.08	*p* = 0.072 *d* = 0.49	*p* = 0.402 *d* = 0.33
Isometric strength	*p* = 0.653 *d* = 0.16	*p* = 0.124 *d* = 0.47	*p* = 0.552 *d* = 0.16	*p* = 0.648 *d* = 0.16	*p* = 0.139 *d* = 0.41	*p* = 0.113 *d* = 0.59	*p* = 0.548 *d* = 0.24	*p* = 0.987 *d* = 0.00

For the magnitude of the changes in KF ROM, the three-way ANOVA only showed main effects for group (*F* = 4.358, *p* = 0.047, and partial *η*^2^ = 0.148) and time (*F* = 22.981, *p* < 0.001, and partial *η*^2^ = 0.479). The pairwise comparison showed significant difference between the CON and EXP groups (overall ΔROM: EXP vs. CON mean ± SD = −20.31 ± 2.64 vs. −12.04 ± 2.95°, *p* = 0.047).

**Figure 4:**
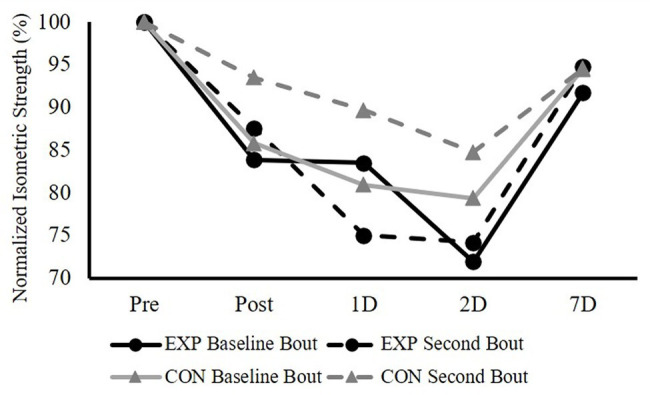
The responses of KF maximal isometric strength (normalized as the percentage of the Pre-testing isometric strength) immediately after (Post), 1 day after (1D), 2 days after (2D), and 7 days after (7D) the baseline and the second bouts of ECC for both EXP and CON groups.

Figure 4 shows both groups’ responses of maximal isometric strength (normalized as the percentage of the Pre-testing isometric strength) following both baseline and second bouts of ECC. For the magnitude of the changes in normalized KF isometric strength, the three-way ANOVA showed a significant bout × group × time (*F* = 3.017, *p* = 0.033, and partial *η*^2^ = 0.109) interaction and a main effect for time (*F* = 17.248, *p* < 0.001, and partial *η*^2^ = 0.408). The follow-up paired samples *t*-tests for the comparisons of the magnitude of changes in KF isometric strength at each time point between the baseline bout and the second bout for both groups did not observe any significant differences (all values of *p* > 0.05; Table 2).

### Index of Protection

At 1D post-exercise, the indices of protection were not significantly different between the EXP and CON groups for both muscle soreness (*p* = 0.696, *d* = 0.15) and ROM (*p* = 0.411, *d* = 0.32). For the KF isometric strength, the indices were significantly different between the EXP and the CON (EXP vs. CON mean ± SD = −29.36 ± 29.21 vs. 55.28 ± 23.83%, *p* = 0.040, *d* = 0.84). At 2D post-exercise, the indices of protection were not significantly different between the EXP and CON groups for all indirect muscle damage markers (muscle soreness: *p* = 0.095, *d* = 0.67; ROM: *p* = 0.250, *d* = 0.46; KF isometric strength: *p* = 0.213, *d* = 0.49).

## Discussion

The purpose of this study was to investigate whether an initial unilateral EF ECC bout would confer a protective effect on muscle damage from performing a subsequent similar ECC in the remote unrelated KF muscle group. The main findings of this study are summarized as follows: (1) The EF ECC bout successfully induced local muscle damage, as evidenced by the prolonged elevations of all the indirect muscle damage markers; (2) For both EXP and CON groups, the magnitude of changes in the KF indirect muscle damage markers between the baseline and the second bouts were not significantly different; and (3) Relative to the CON, adding the EF ECC bout compromised the recovery of the KF isometric strength following the subsequent KF ECC bout, particularly at the 1-day post-exercise time point. Overall, these results did not provide evidence for the non-local RBE. Instead, part of our pilot data suggested an exacerbating effect on the eccentric exercise-induced muscle damage (e.g., muscular strength), if a prior ECC bout is performed in a remote unrelated muscle group.

The initial arm eccentric exercise-induced local indirect muscle damage markers’ response patterns and ranges were generally in agreement with the ones from some previous studies (Chen et al., 2016, 2019). Except for the ROM, other EF indirect muscle damage markers were not back to the baseline, even 7 days after the ECC intervention. With the muscle-damaging stimuli from the unilateral EF muscles, previous research has demonstrated RBE from the contralateral homologous muscle (Chen et al., 2016). Because the contralateral muscle group is not exposed to the initial bout of ECC, the only plausible mechanisms are limited to neural and inflammatory adaptations. As mentioned, the attenuation of the inflammatory responses following the second ECC bout in the contralateral muscle could be regulated through neural pathway signaling, or through the circulation. If the circulating factor plays a role, then not only the homologous contralateral muscle, but all muscles would be conferred the protective effect. Thus, our pilot work aimed to examine if such damaging stimuli could confer any protective effect on the subsequent eccentric exercise-induced muscle damage in the non-local KF muscles. To our knowledge, this is the first study to examine the potential non-local RBE between remote unrelated muscle groups. Our results showed that, for the EXP group, all KF muscle damage indirect markers’ responses following the second KF ECC bout were not significantly different from the ones following the baseline KF ECC.

It is interesting to note that the EXP group showed a medium effect (at 2D: *d* = 0.52; and 7D: *d* = 0.54) for the attenuation of the magnitude of the increased the KF muscle soreness response following the ECC. Additionally, this was also accompanied with the medium effect for the group difference (*p* = 0.095, *d* = 0.67) for the index of protection at 2D for the muscle soreness. However, this does not necessarily indicate a potential non-local RBE. Because other muscle damage indirect markers in the current study did not show the similar pattern. Additionally, muscle soreness rating alterations may potentially be due to an increased pain tolerance, which can be centrally mediated. For example, by having participants do unilateral and contralateral step-down (eccentric) exercise separated by 2 weeks, the very first study to examine the CL-RBE found significant attenuation in pain scores, but not in the muscle strength loss and muscle tenderness, following the contralateral ECC bout (Connolly et al., 2002). The authors attributed the changes in muscle pain to the subjects being more familiar with the testing related discomfort from experiencing the pain with the initial bout, rather than a real CL-RBE.

Regarding the KF isometric strength, the responses of strength changes following ECC bouts and the indices of protection for both groups showed some interesting patterns. At 1-day post-exercise, the indices of protection were significantly different between groups, with CON showing a positive protective percentage (55.28 ± 23.83%), but negative percentage for the EXP (−29.36 ± 29.21%), indicating that adding a prior EF ECC bout might have exacerbating effect on the KF strength loss following its ECC. As for the CON, even though the baseline and second ECC bouts were 6 weeks apart, it is interesting that our data showed a medium effect (*d* = 0.59) for the CL-RBE. We chose to separate the two KF ECC bouts by 6 weeks, because Chen et al. (2018) showed that the KF CL-RBE disappeared between 7 and 28 days. Additionally, our CON group’s index of protection seems to be greater than those from Chen et al. (2018). The different findings between the current study and Chen et al. (2018) regarding the CL-RBE could be due to the different ECC protocols, where maximal isokinetic ECC was used in that study. A surprising finding for the EXP is that, relative to the CON, the KF isometric strength had a negative index of protection percentage. It is also worth mentioning that the SD for this index is 29.21%, indicating a large inter-subject variability. It is not clear what potential underlying mechanisms can be for this “non-local exacerbating effect,” but the current findings do not support the evidence for the non-local RBE.

Even the current study provided some novel and interesting findings, it is important to mention that this investigation does have a major limitation. Specifically, the current pilot study only examined a few indirect muscle damage markers, but some other biochemical (e.g., creatine kinase and myoglobin) and molecular (e.g., nuclear-factor kappa B) markers were not measured. Even though the current results do not support the role of circulating factor on the potential inflammatory response mechanism of the RBE, without directing measuring these markers, a clear conclusion still cannot be made.

In conclusion, after the initial unilateral elbow flexion (EF) ECC bout, RBE is not evident in the non-local unrelated lower limb knee flexor muscles, even though there was a medium effect for the attenuation of the increased knee flexor muscle soreness level. Additionally, the isometric strength response in the knee flexor muscles was worsened, suggesting that instead of a protective effect, an exacerbating damaging effect on the non-local unrelated muscles can be expected. While it is unclear what the potential mechanisms might be for this phenomenon, this information can be useful in some practical and/or clinical situations: practitioners need to be aware that an initial upper limb muscle high-intensity ECC bout may make limb muscles more susceptible to eccentric exercise-induced muscle damage, thus affecting muscle recovery. Therefore, this needs to be taken into consideration when designing training and rehabilitation programs.

## Data Availability Statement

The raw data supporting the conclusions of this article will be made available by the authors, without undue reservation.

## Ethics Statement

The studies involving human participants were reviewed and approved by University of Mississippi Institutional Review Board. The patients/participants provided their written informed consent to participate in this study.

## Author Contributions

XY, WM, and SJ contributed to the design of the work. XY, WM, SJ, JS, and TW contributed to the data acquisition, analysis, and interpretation of data. XY, WM, SJ, and JS contributed to the draft of the work and revised it critically for important intellectual content. XY, WM, SJ, JS, and TW approved the version to be submitted, and agreed to be accountable for all aspects of the work in ensuring that the questions related to the accuracy or integrity of any part of the work are appropriately investigated and resolved. All authors contributed to the article and approved the submitted version.

### Conflict of Interest

The authors declare that the research was conducted in the absence of any commercial or financial relationships that could be construed as a potential conflict of interest.

## References

[ref1] Beck T. W. (2013). The importance of a priori sample size estimation in strength and conditioning research. J. Strength Cond. Res. 27, 2323–2337. 10.1519/JSC.0b013e318278eea0, PMID: 23880657

[ref2] Beck T. W. Ye X. Wages N. P. (2016). Differential effects of unilateral concentric vs. eccentric exercise on the dominant and nondominant forearm flexors. J. Strength Cond. Res. 30, 703–709. 10.1519/JSC.0000000000001137, PMID: 26907841

[ref3] Behm D. G. Alizadeh S. Anvar S. H. Drury B. Granacher U. Moran J. (2021). Non-local acute passive stretching effects on range of motion in healthy adults: a systematic review with meta-analysis. Sports Med. 10.1007/s40279-020-01422-5, PMID: [Epub ahead of print] 33459990

[ref4] Borg G. (1998). Borg's perceived exertion and pain scales. Champaign, IL, USA: Human kinetics.

[ref5] Brandenberger K. J. Warren G. L. Ingalls C. P. Otis J. S. Doyle J. A. (2019). Downhill running impairs activation and strength of the elbow flexors. J. Strength Cond. Res. 10.1519/JSC.0000000000003111, PMID: [Epub ahead of print] 30908371

[ref6] Chen T. C. Chen H. L. Cheng L. F. Chou T. Y. Nosaka K. (2021). Effect of leg eccentric exercise on muscle damage of the elbow flexors after maximal eccentric exercise. Med. Sci. Sports Exerc. 10.1249/MSS.0000000000002616, PMID: [Epub ahead of print] 33560777

[ref7] Chen T. C. Chen H. L. Lin M. J. Yu H. I. Nosaka K. (2016). Contralateral repeated bout effect of eccentric exercise of the elbow flexors. Med. Sci. Sports Exerc. 48, 2030–2039. 10.1249/MSS.0000000000000991, PMID: 27187096

[ref8] Chen T. C. Lin M. J. Chen H. L. Yu H. I. Nosaka K. (2018). Contralateral repeated bout effect of the knee flexors. Med. Sci. Sports Exerc. 50, 542–550. 10.1249/MSS.0000000000001470, PMID: 29077637

[ref9] Chen T. C. Yang T. J. Huang M. J. Wang H. S. Tseng K. W. Chen H. L.. (2019). Damage and the repeated bout effect of arm, leg, and trunk muscles induced by eccentric resistance exercises. Scand. J. Med. Sci. Sports 29, 725–735. 10.1111/sms.13388, PMID: 30663816

[ref10] Cohen J. (1988). Statistical power analysis for the behavioral sciences. 2nd Edn. Hillsdale, NJ: Lawrence Earlbaum Associates.

[ref11] Connolly D. A. J. Reed B. Mchugh M. P. (2002). The repeated bout effect: does evidence for a crossover effect exist? J. Sports Sci. Med. 1, 80–86. PMID: 24701128 PMC3967433

[ref12] Doix A. M. Wachholz F. Marterer N. Immler L. Insam K. Federolf P. A. (2018). Is the cross-over effect of a unilateral high-intensity leg extension influenced by the sex of the participants? Biol. Sex Differ. 9:29. 10.1186/s13293-018-0188-4, PMID: 29954447 PMC6022493

[ref13] Halperin I. Chapman D. W. Behm D. G. (2015). Non-local muscle fatigue: effects and possible mechanisms. Eur. J. Appl. Physiol. 115, 2031–2048. 10.1007/s00421-015-3249-y, PMID: 26330274

[ref14] Hopkins W. G. (2000). Measures of reliability in sports medicine and science. Sports Med. 30, 1–15. 10.2165/00007256-200030010-00001, PMID: 10907753

[ref15] Howatson G. Taylor M. B. Rider P. Motawar B. R. Mcnally M. P. Solnik S.. (2011). Ipsilateral motor cortical responses to TMS during lengthening and shortening of the contralateral wrist flexors. Eur. J. Neurosci. 33, 978–990. 10.1111/j.1460-9568.2010.07567.x, PMID: 21219480 PMC3075420

[ref16] Howatson G. van Someren K. A. (2007). Evidence of a contralateral repeated bout effect after maximal eccentric contractions. Eur. J. Appl. Physiol. 101, 207–214. 10.1007/s00421-007-0489-5, PMID: 17534644

[ref17] Hyldahl R. D. Chen T. C. Nosaka K. (2017). Mechanisms and mediators of the skeletal muscle repeated bout effect. Exerc. Sport Sci. Rev. 45, 24–33. 10.1249/JES.0000000000000095, PMID: 27782911

[ref18] Jeon S. Miller W. M. Kang M. Ye X. (2019). The minimum number of attempts for a reliable isometric strength test score. J. Sci. Sport Exerc. 2, 89–95. 10.1007/s42978-019-00035-3

[ref19] Kidgell D. J. Frazer A. K. Daly R. M. Rantalainen T. Ruotsalainen I. Ahtiainen J.. (2015). Increased cross-education of muscle strength and reduced corticospinal inhibition following eccentric strength training. Neuroscience 300, 566–575. 10.1016/j.neuroscience.2015.05.057, PMID: 26037804

[ref20] Killen B. S. Zelizney K. L. Ye X. (2019). Crossover effects of unilateral static stretching and foam rolling on contralateral hamstring flexibility and strength. J. Sport Rehabil. 28, 533–539. 10.1123/jsr.2017-0356, PMID: 29543123

[ref21] Miller W. Kang M. Jeon S. Ye X. (2019). A meta-analysis of non-local heterologous muscle fatigue. J. Trainol. 8, 9–18. 10.17338/trainology.8.1_9

[ref22] Nosaka K. Clarkson P. M. (1995). Muscle damage following repeated bouts of high force eccentric exercise. Med. Sci. Sports Exerc. 27, 1263–1269. PMID: 8531624

[ref23] Nosaka K. Sakamoto K. E. I. Newton M. Sacco P. (2001). How long does the protective effect on eccentric exercise-induced muscle damage last? Med. Sci. Sports Exerc. 33, 1490–1495. 10.1097/00005768-200109000-00011, PMID: 11528337

[ref24] Starbuck C. Eston R. G. (2012). Exercise-induced muscle damage and the repeated bout effect: evidence for cross transfer. Eur. J. Appl. Physiol. 112, 1005–1013. 10.1007/s00421-011-2053-6, PMID: 21720885

[ref25] Tsuchiya Y. Nakazato K. Ochi E. (2018). Contralateral repeated bout effect after eccentric exercise on muscular activation. Eur. J. Appl. Physiol. 118, 1997–2005. 10.1007/s00421-018-3933-9, PMID: 29987366

[ref26] Warren G. L. Lowe D. A. Armstrong R. B. (1999). Measurement tools used in the study of eccentric contraction-induced injury. Sports Med. 27, 43–59. 10.2165/00007256-199927010-00004, PMID: 10028132

[ref27] Weir J. P. (2005). Quantifying test-retest reliability using the intraclass correlation coefficient and the SEM. J. Strength Cond. Res. 19, 231–240. 10.1519/15184.1, PMID: 15705040

[ref28] Xin L. Hyldahl R. D. Chipkin S. R. Clarkson P. M. (2014). A contralateral repeated bout effect attenuates induction of NF-kappaB DNA binding following eccentric exercise. J. Appl. Physiol. 116, 1473–1480. 10.1152/japplphysiol.00133.2013, PMID: 23950163

[ref29] Ye X. Beck T. W. Defreitas J. M. Wages N. P. (2014). An examination of the strength and electromyographic responses after concentric vs. eccentric exercise of the forearm flexors. J. Strength Cond. Res. 28, 1072–1080. 10.1519/JSC.0000000000000251, PMID: 24077382

[ref30] Ye X. Beck T. W. Wages N. P. (2015). Acute effects of concentric vs. eccentric exercise on force steadiness and electromyographic responses of the forearm flexors. J. Strength Cond. Res. 29, 604–611. 10.1519/JSC.0000000000000674, PMID: 25226337

[ref31] Ye X. Beck T. W. Wages N. P. Carr J. C. (2018). Sex comparisons of non-local muscle fatigue in human elbow flexors and knee extensors. J. Musculoskelet. Neuronal Interact. 18, 92–99. PMID: 29504584 PMC5881134

